# [−2]proPSA versus ultrasensitive PSA fluctuations over time in the first year from radical prostatectomy, in an high-risk prostate cancer population: A first report

**DOI:** 10.1186/s12894-016-0131-0

**Published:** 2016-03-24

**Authors:** S. De Luca, R. Passera, A. Sottile, C. Fiori, R. M. Scarpa, F. Porpiglia

**Affiliations:** Division of Urology, San Luigi Gonzaga Hospital and University of Torino, Orbassano, Italy; Division of Nuclear Medicine, San Giovanni Battista Hospital and University of Torino, Corso AM Dogliotti 14, 10126 Torino, Italy; Division of Laboratory Medicine, Candiolo Cancer Institute, Candiolo, Italy

**Keywords:** Prostate cancer, (−2)pro-prostate-specific antigen, Prostate-specific antigen, Biochemical recurrence

## Abstract

**Background:**

[−2]proPSA and its derivatives have an higher diagnostic accuracy than PSA in predicting prostate cancer (PCa). In alternative to PSA, ultrasensitive PSA (uPSA) and [−2]proPSA could be potentially useful in recurrent disease detection. This research focused on [−2]proPSA and uPSA fluctuations over time and their possible clinical and pathological determinants, in the first year after RP.

**Methods:**

A cohort of 106 consecutive patients, undergoing RP for high-risk prostate cancer (pT3/pT4 and/or positive margins), was enrolled. No patient received either preoperative/postoperative androgen deprivation therapy or immediate adjuvant RT, this latter for patient choice. [−2]proPSA and uPSA were measured at 1, 3, 6, 9, 12 months after RP; their trends over time were estimated by the mixed-effects linear model. The uPSA relapse was defined either as 3 rising uPSA values after nadir or 2 consecutive uPSA >0.2 ng/ml after RP.

**Results:**

The biochemical recurrence (BCR) rate at 1 year after RP was either 38.6 % (in case of 3 rising uPSA values) or 34.9 % (in case of PSA >0.2 ng/ml after nadir), respectively. The main risk factors for uPSA fluctuations over time were PSA at diagnosis >8 ng/ml (*p* = 0.014), pT (*p* = 0.038) and pN staging (*p* = 0.001). In turn, PSA at diagnosis >8 ng/ml (*p* = 0.012) and pN (*p* < 0.001) were the main determinants for [−2]proPSA trend over time. In a 39 patients subgroup, uPSA decreased from month 1 to 3, while [−2]proPSA increased in 90 % of them; subsequently, both uPSA and [−2]proPSA increased in almost all cases. The [−2]proPSA trend over time was independent from BCR status either in the whole cohort as well in the 39 men subgroup.

**Conclusions:**

Both uPSA and [−2]proPSA had independent significant fluctuations over time. PSA at diagnosis >8 ng/ml and pathological staging significantly modified both these trends over time. Since BCR was not confirmed as determinant of [−2]proPSA fluctuations, its use as marker of early biochemical relapse may not be actually recommended, in an high-risk prostate cancer patients population.

## Background

After a successful radical prostatectomy (RP), a serum detectable Prostate Specific Antigen (PSA) may be considered a marker of residual prostate tissue, presumably anticipating either locoregional or systemic disease [1].

However, approximately 20 % of patients experience biochemical recurrence (BCR) after RP [2–4]; around 30 % of them will ultimately develop a clinical progression [5, 6].

Several trials proved that adjuvant radiation therapy (RT) after RP decreases BCR risk and provides survival benefit, in high-risk disease patients. An early and reliable BCR detection is therefore crucial, since postoperative RT is more effective when given to subjects having low PSA levels [7].

New PSA ultrasensitive methods detect levels <0.1 ng/ml, and some assays even minimal ones (1 pg/ml) [8, 9]. A classical definition for ultrasensitive PSA (uPSA) relapse is 3 rising uPSA values after nadir [10]; recurrence seldom occurs in patients with uPSA <0.04 ng/ml, 3 years after RP [11].

In recent years, many efforts were made to improve the biomarkers diagnostic accuracy for prostate cancer (PCa); at the same time, an alternative to PSA as BCR marker is still unavailable. Some studies showed that [−2]proPSA and its derivatives improve PSA accuracy in predicting PCa at prostate biopsy (Bx), being associated to PCa aggressiveness either at Bx or at final pathology after RP [12, 13]. Moreover, [−2]proPSA could be potentially useful in recurrent disease detection, a virtually unexplored field.

To our knowledge, no study investigated the [−2]proPSA trend over time post-RP. We enrolled at RP a sequential cohort of high-risk PCa patients (extra prostate disease and/or positive margins), eligible for adjuvant RT but not being given for patient choice.

The primary endpoint of this research was to longitudinally investigate either [−2]proPSA and uPSA time trends as well their clinical and pathological determinants, in the first year after RP, at a single high-volume institution. The secondary endpoint was to elucidate a [−2]proPSA possible role in early BCR detection.

## Methods

A cohort of 106 consecutive patients, undergoing robot assisted radical prostatectomy (RARP) for a pathological high-risk PCa (pT3/pT4 and/or positive margins), was enrolled at San Luigi Gonzaga Hospital - Orbassano (Italy) from September 2013 to October 2014. Among them, 83 patients (81.3 %) underwent robot-assisted extended pelvic lymph nodes dissection prior to RARP (external iliac artery/vein, obturator fossa, obturator nerve, internal iliac artery/presacral lymph nodes) [14]. Lymphadenectomy was planned according to Briganti nomogram [15]. Pathological staging was performed according to the TNM Classification of Malignant Tumors seventh edition [16]; histological grading was assessed according to the Gleason grading system [17]. No patient received either preoperative/postoperative androgen deprivation therapy or immediate adjuvant RT, this latter for patient choice.

The follow-up was scheduled at 1/3/6/9/12 months after RARP; it included a complete physical and digital rectal examination (DRE), as like [−2]proPSA and uPSA measurements. The uPSA relapse was defined either as 3 rising uPSA values after nadir [10] or two consecutive uPSA >0.2 ng/ml rising after RP [18].

Due to the observational nature of this research and according to Italian regulation, no formal IRB/IEC approval was needed [19].

### uPSA and [−2]proPSA serum concentrations measurement

Serum samples were analysed by the Access 2 Immunoassay System on a UniCell DxI800 instrument (Beckman Coulter, USA). The calibration procedure was performed by a 7-point recombinant [−2]proPSA curve (0–5000 pg/ml). The blank and quantitation limits (according to the Clinical Laboratory Standards Institute document EP17-A) were 0.5 and 3.23 pg/ml, respectively. uPSA results were obtained by a single determination, while that from [−2]proPSA by a duplicate one; the analyses were repeated in case of coefficient of variation >20 %. All analyses were performed in the same laboratory (Candiolo Cancer Institute).

### Statistical methods

The primary outcomes were the uPSA and [−2]proPSA trends over time after RARP and their potential modifications by independent covariates. uPSA and [−2]proPSA were longitudinally measured at five time-points (1/3/6/9/12 months after RP), and these repeated measures were used as dependent variables in univariate and multivariate mixed-effects linear models [20]. Due to the not-Gaussian distribution of uPSA and [−2]proPSA, all models were estimated using their log-transformed values [ln(uPSA) and ln [−2]proPSA]. At first, the univariate analyses were performed for the following covariates: age (>65 vs. ≤65 years), Body Mass Index [BMI, (>26 vs. ≤26)], DRE (positive vs. negative), PSA at diagnosis (>8 vs. ≤8 ng/ml), number of positive Bx samples (>5 vs. ≤5), GS at Bx (8–9 vs. ≤7), number of lesions at Magnetic Resonance Imaging [MRI (≥2 vs. 1)], prostate volume (>40 vs. ≤40 ml), tumor percentage (>10 % vs. ≤10 %), GS at surgery (8–9 vs. ≤7), capsule/vesicles/neural/vascular/marginal involvement (any vs. none), pT (pT3b vs. pT3a vs. pT2), pN (pN+ vs. pN0) and BCR (any vs. none). Two different definitions of BCR were used: either uPSA value >0.2 ng/ml or 3 rising uPSA values after nadir. The multivariate mixed-effects linear models for uPSA and [−2]proPSA trends over time were estimated by the restricted maximum likelihood method, using a first-order autoregressive covariance matrix: both ln(uPSA) and ln([−2]proPSA) variances at each time-point were considered comparable and constant, while the correlations between subsequent measures similar. Patient characteristics were analyzed by the Fisher’s exact test for categorical variables, while for continuous ones by the Mann–Whitney and Kruskal-Wallis tests (for independent measures) or by the Wilcoxon and Friedman ones (for repeated measures). All results for continuous variables were expressed as the median (range). All reported p-values were obtained by the two-sided exact method, at the conventional 5 % significance level. Data were analysed as of September 2015 by R 3.2.1 (R Foundation for Statistical Computing, Vienna-A, http://www.R-project.org).

## Results

The main patient characteristics (106 patients) are reported in Table 1. The 1-year after surgery BCR rate was 34.9 % using uPSA value > 0.2 ng/ml, while 38.6 % using 3 rising uPSA values after nadir; at the same time, no subject had an imaging-confirmed metastatic disease.

**Table 1:** Main patient characteristics

Age at diagnosis, years	65 (48–77)
PSA at diagnosis, ng/ml	7.8 (4.0–81.0)
BMI at diagnosis, kg/m^2^	26.3 (17.4–34.6)
Prostate volume, ml	41.2 (22.4–103.9)
Tumor percentage, %	10.2(2.3–52.9)
Cancer familiarity,	
neg	6 (5.7 %)
pos	100 (94.3 %)
DRE,	
neg	71 (67.6 %)
pos	34 (32.4 %)
GS at biopsy,	
6	31(29.5 %)
7(3 + 4)	29(27.6 %)
7(4 + 3)	21(20.0 %)
8	16(15.2 %)
9	8(7.6 %)
Lesions at MRI,	
1	31 (53.4 %)
2+	27 (46.6 %)
GS at surgery,	
6	4(3.8 %)
7(3 + 4)	37(37.4 %)
7(4 + 3)	37(37.4 %)
8	15(15.2 %)
9	6(6.1 %)
Margins,	
neg	53 (50.0 %)
pos	53 (50.0 %)
Capsule involvement,	
neg	25 (23.6 %)
pos	81 (76.4 %)
Neural involvement,	
neg	8 (7.5 %)
pos	98 (92.5 %)
Vascular involvement,	
neg	65 (68.4 %)
pos	30 (31.6 %)
pT2	9 (8.5 %)
pT3a	83 (78.3 %)
pT3b	14 (13.2 %)
pN0	69 (79.3 %)
pN+	18 (20.7 %)
uPSA, ng/ml 1/3/6/9/12 months	0.010 (0–1.15)/0.018 (0–0.67)/0.051 (0.01–0.52)/0.100 (0.01–0.56)/0.154 (0–0.89)
[−2]proPSA, pg/ml 1/3/6/9/12 months	0.22 (0–2.14)/0.57 (0–2.84)/0.89 (0–3.97)/1.25 (0.03–3.72)/1.38 (0–4.86)

For continuous variables, all the results are expressed as median (range)

The uPSA values sequentially increased in 43.4/69.8/83.1/89.0 % patients, at 3/6/9/12 months after RARP, respectively. The uPSA trend over time was confirmed by the Friedman test (*p* < 0.001); using the Wilcoxon one, all the differences between two adjacent time-points were extremely significant (*p* < 0.001), except that between 1 vs. 3 months (*p* = 0.833).

**Fig. 1:**
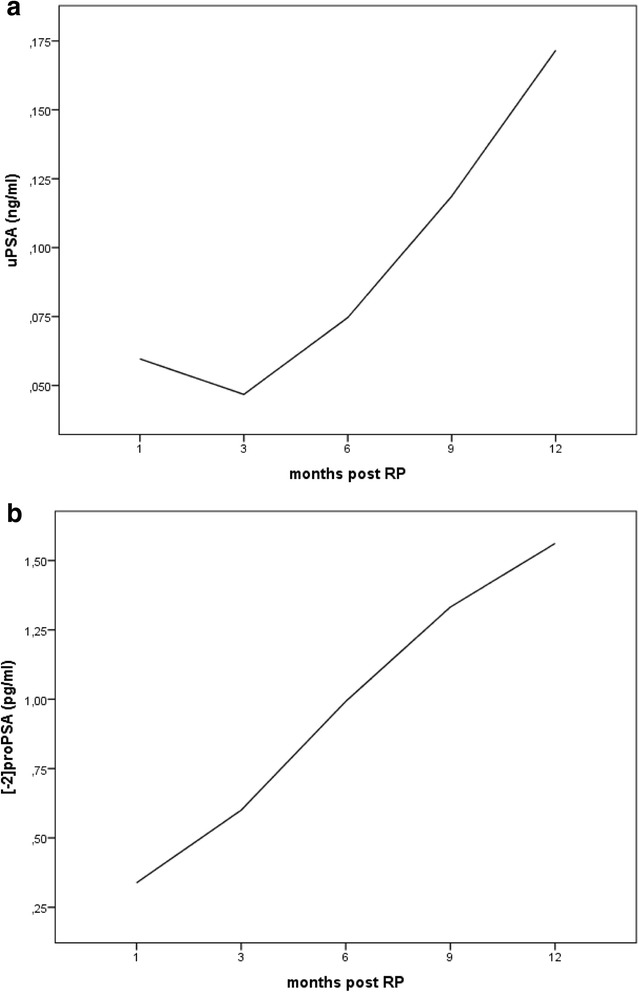
**a**, **b** uPSA and [−2]proPSA mean observed values at 1–3–6–9–12 months after radical prostatectomy

The mixed-effects linear model was used to confirm the uPSA time trend (within-subject factor) and to estimate its potential risk factors (between-subject factors) (Table 2, Fig. 1a). This approach confirmed that uPSA fluctuations over time were statistically significant (*p* < 0.001). In the multivariate mixed-effects linear model, the main determinants for uPSA fluctuations were PSA at diagnosis >8 ng/ml (*p* = 0.014), pT (*p* = 0.038) and pN (*p* = 0.001).

**Table 2:** Mixed linear models for uPSA and [−2]proPSA repeated measures

uPSA	Univariate model	Multivariate model	[−2]proPSA	Univariate model	Multivariate model
Covariate	*p*	*p*	Covariate	*p*	*p*
Time trend	**<0.001**	**<0.001**	Time trend	**<0.001**	**<0.001**
Age >65 years	0.095	0.455	Age >65 years	0.472	
BMI >26	0.059	0.507	BMI >26	0.315	
PSA at diagnosis >8 ng/ml	**0.015**	**0.014**	PSA at diagnosis >8 ng/ml	**0.032**	**0.012**
Positive DRE	0.168		Positive DRE	0.241	
Biopsy samples >5	0.365		Biopsy samples >5	0.483	
GS at biopsy >7	0.419		GS at biopsy >7	0.349	
MRI lesions >1	0.178		MRI lesions >1	0.607	
Prostate volume >40 ml	0.214		Prostate volume >40 ml	0.472	
Tumor percentage >10 %	0.522		Tumor percentage >10 %	0.658	
Positive margins	**0.018**	0.214	Positive margins	0.822	
GS at surgery >7	0.209		GS at surgery >7	0.102	
Capsule involvement	**0.039**		Capsule involvement	0.077	
Neural involvement	0.216		Neural involvement	0.603	
Vascular involvement	0.110		Vascular involvement	0.083	0.952
pT	**<0.001**	**0.038**	pT	**0.008**	0.879
pN	**<0.001**	**0.001**	pN	**0.008**	**<0.001**
			BCR (uPSA +0,2 ng/ml)	0.096	
			BCR (3 rising uPSA values)	0.194	
pT*pN (interaction)	-	0.639	PSA at diagnosis*pN (interaction)	-	0.099

*p*-values

The [−2]proPSA values sequentially increased in 86.8/93.4/90.4/91.5 % patients, at 3/6/9/12 months after surgery, respectively. The difference among the median [−2]proPSA repeated measures was highly significant at the Friedman test (*p* < 0.001); all the differences between adjacent time-points were extremely significant (*p* < 0.001), when investigated by the Wilcoxon test.

Using the mixed-effects linear model as for uPSA, [−2]proPSA fluctuations over time were statistically significant (*p* < 0.001) (Table 2, Fig. 1b). Their main predictors were PSA at diagnosis >8 ng/ml (*p* = 0.012) and pN (*p* < 0.001); the interaction between them was marginal (*p* = 0.099).

In 39 patients, uPSA decreased from month 1 to 3, conversely [−2]proPSA increased in 90 % of them; in the further follow-up, both uPSA and [−2]proPSA increased in almost all cases.

**Fig. 2:**
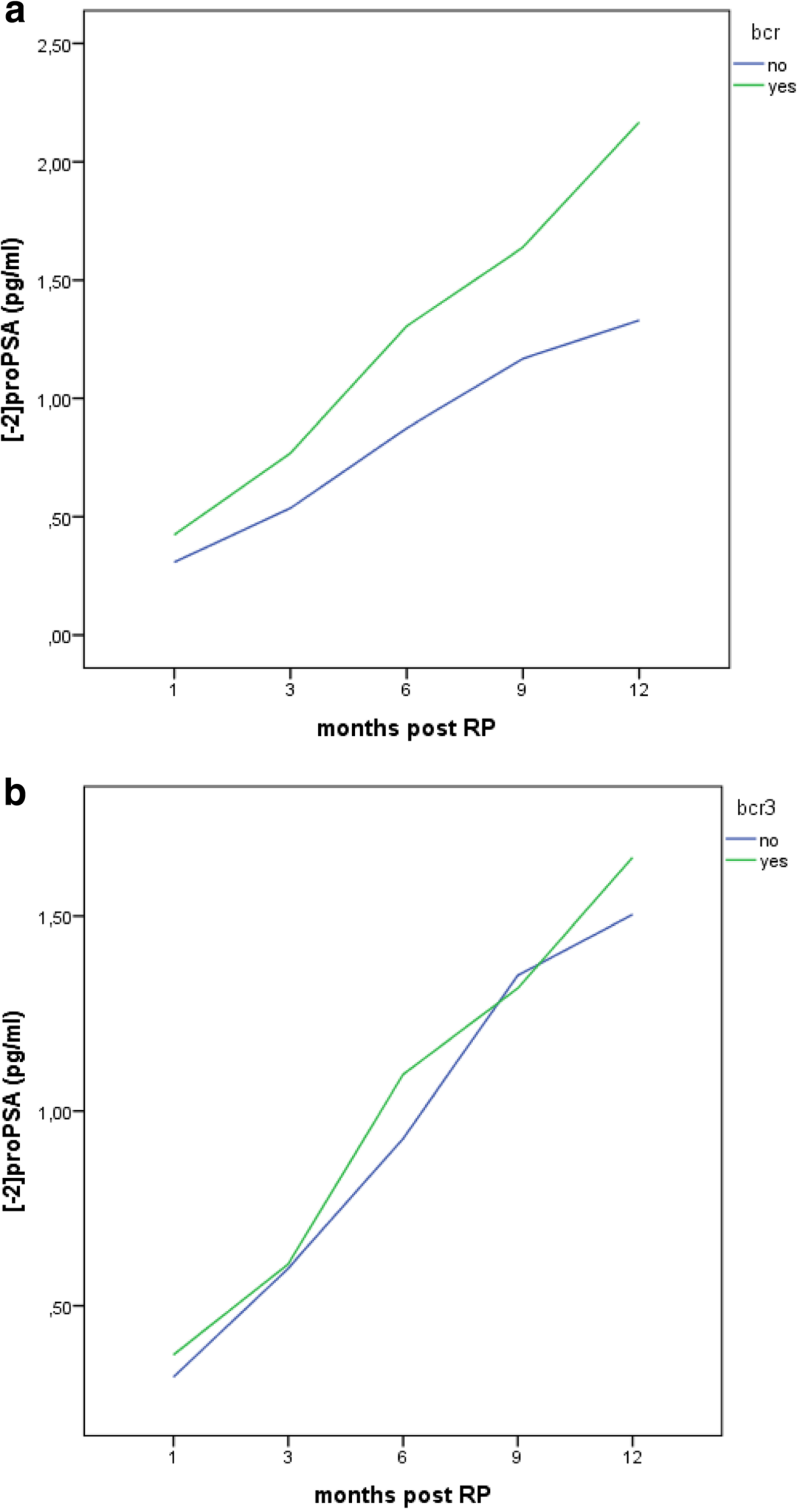
**a**, **b** [−2]proPSA mean observed values at 1–3–6–9–12 months after radical prostatectomy by both BCR definitions (bcr = uPSA value increasing 0.2 ng/ml; bcr3 = 3 rising uPSA values after nadir)

Of note, [−2]proPSA trend over time was independent from BCR status (*p* = 0.096 and 0.194 according to BCR definitions, respectively; Fig. 2a, b) in the whole cohort as well in the 39 men subgroup (its BCR rate was 33.3 %). When BCR was calculated as uPSA >0.2 ng/ml, the 1-year [−2]proPSA was around double for BCR patients compared to no-BCR ones (its observed marginal means were 0.31–0.54–0.87–1.17–1.33 pg/ml for BCR subjects and 0.41–0.77–1.31–1.64–2.17 pg/ml for no-BCR ones). When BCR was calculated as 3 rising uPSA values after nadir, [−2]proPSA increased over time with a superimposable pattern (0.31–0.60–0.93–1.35–1.50 pg/ml for BCR subjects and 0.37–0.61–1.09–1.32–1.65 pg/ml for no-BCR ones).

## Discussion

Recently, while many efforts have been made to improve the biomarkers diagnostic accuracy for PCa, an alternative to PSA as BCR marker is still lacking.

A persistently elevated or rising PSA after RP identifies an heterogeneous patient population, having highly variable prognosis and controversial management. Several trials showed that adjuvant RT after RP decreases BCR risk and provides survival benefit for high-risk patients [21–23]. Conversely, surgical treatment would not have failed in up to 50 % of patients receiving adjuvant RT: thus, they would have been unnecessarily exposed to radiations. To minimize overtreatment, RT should be reserved only for confirmed recurrences; however, an early salvage RT has not been proven to be equivalent to adjuvant RT yet.

uPSA is an interesting tool, whose variations could foresee a BCR [11]; this finding rarely occurs in patients with very low uPSA nadir after RP [24]. However, the uPSA role after RP is not fully defined yet. Recently, Seikkula investigated uPSA after RP and the possible correlation of uPSA doubling time (uPSA-DT) with traditional PSA doubling time (PSA-DT); uPSA >0.03 ng/ml emerged as good relapse predictor, while the above correlation between was marginal [25].

Therefore, new biomarkers for risk stratification after RP are required. Several studies demonstrated that the [−2]proPSA truncated form is detectable in tumor extracts and its serum values are markedly associated with PCa [26–30]. Sokoll demonstrated that [−2]proPSA may improve PCa detection: its raising is associated with an aggressive disease [31]. Le showed that this biomarker was able to discriminate PCa from benign disease, in males with PSA 2.5–10 ng/ml and negative DRE [27]. Recently, Guazzoni confirmed that [−2]proPSA and its derivatives are associated with PCa volume and aggressiveness [13].

Nevertheless, well considering that [−2]proPSA and its derivatives have an higher diagnostic performance than PSA, the potential usefulness of this PSA isoform in the detection of recurrent disease post radical treatment is still quite unexplored.

Sottile investigated the role of [−2]proPSA in the identification of patients with metastatic progression after RP [32]. In this study, 76 patients with BCR were retrospectively studied; the imaging performed at BCR time confirmed metastatic disease in 31 out of them. Serum samples were collected at the time of imaging-confirmed metastatic progression. Median PSA, free PSA (fPSA), %fPSA, [−2]proPSA and PHI were compared between metastatic and non-metastatic patients; [−2]proPSA was a statistically significant predictor of imaging-proven metastatic PCa. However, [−2]proPSA was assessed only at BCR time, so no information may be derived on its potential role, in predicting subsequent clinical progression, when measured at BCR time [32].

The current trial is the first investigating [−2]proPSA fluctuations over time post-RP, and their possible determinants in an high-risk PCa patients cohort.

The [−2]proPSA time trend in the first 3 months showed a different pattern, compared to uPSA one. While uPSA had quite stable levels in two thirds of cases (slowly increasing only in the next period), [−2]proPSA showed a constant linear increase.

The main risk factors for uPSA fluctuations were PSA at diagnosis, pT and pN staging, being PSA at diagnosis and pN the only ones for [−2]proPSA variations.

A secondary endpoint was to investigate [−2]proPSA potential role as BCR early biomarker, in comparison to uPSA: in our series, the [−2]proPSA trend over time was independent from BCR status.

Our study has some points of strengths. The main one is that our results may suggest a stop in further researches for [−2]proPSA in the post RP arena. Second, it was designed as observational study in a homogeneous cohort of men with high-risk PCa, candidates for RP. Finally, we adopted a standardized centralised pathological evaluation; all blood samples were managed in the same laboratory according to Semjonow guidelines: no archived serum were used [33].

## Conclusions

[−2]proPSA and uPSA showed significant fluctuations over time after RP, with an independent pattern. PSA at diagnosis and pathological staging significantly modified both these trends. Since BCR was not confirmed as a modifier of [−2]proPSA time trend, its use as marker of an early biochemical relapse may not be actually recommended, among high-risk prostate cancer patients.

### Ethics approval and consent to participate

Due to the observational nature of this research and according to Italian regulation, the San Luigi Gonzaga Hospital (Orbassano-Italy) IRB/IEC was notified about this research proposal, while no formal ethics approval was needed [19].

## Abbreviations

[−2]proPSA: [−2]pro-prostate specific antigen
BCR: biochemical recurrence
BMI: Body Mass Index
Bx: biopsy
DRE: digital rectal examination
DT: doubling time
GS: Gleason score
LNI: lymph nodes invasion
MRI: magnetic resonance imaging
PCa: prostate cancer
PSA: prostate specific antigen
RARP: robot assisted radical prostatectomy
RP: radical prostatectomy
RT: radiotherapy
uPSA: ultrasensitive prostate specific antigen
